# Chicken Essence for Cognitive Function Improvement: A Systematic Review and Meta-Analysis

**DOI:** 10.3390/nu8010057

**Published:** 2016-01-20

**Authors:** Siew Li Teoh, Suthinee Sudfangsai, Pisake Lumbiganon, Malinee Laopaiboon, Nai Ming Lai, Nathorn Chaiyakunapruk

**Affiliations:** 1 School of Pharmacy, Monash University Malaysia, Selangor 47500, Malaysia; charmaine.slteoh@gmail.com; 2 Faculty of Pharmaceutical Sciences, Naresuan University, Phitsanulok 65000, Thailand; suthinee_sfs@hotmail.com; 3 Department of Obstetrics and Gynaecology, Faculty of Medicine, Khon Kaen University, Khon Kaen 40002, Thailand; pisake@kku.ac.th; 4 Department of Biostatistics and Demography, Faculty of Public Health, Khon Kaen University, Khon Kaen 40002, Thailand; laopaiboonmalinee@yahoo.co.uk; 5 School of Medicine, Taylor’s University Malaysia, Selangor 47500, Malaysia; lainm123@yahoo.co.uk; 6 Center of Pharmaceutical Outcomes Research (CPOR), Department of Pharmacy Practice, Faculty of Pharmaceutical Sciences, Naresuan University, Phitsanulok 65000, Thailand; 7 School of Pharmacy, University of Wisconsin, Madison, WI 53705-2222, USA; 8 School of Population Health, University of Queensland, Brisbane, Herston QLD 4006, Australia

**Keywords:** chicken essence, chicken extract, cognitive function, executive function, attention, nutritional product, supplement, health claims

## Abstract

Chicken essence (CE) is a popular traditional remedy in Asia, which is believed to improve cognitive functions. CE company claimed that the health benefits were proven with research studies. A systematic review was conducted to determine the cognitive-enhancing effects of CE. We systematically searched a number of databases for randomized controlled trials with human subjects consuming CE and cognitive tests involved. Cochrane’s Risk of Bias (ROB) tool was used to assess the quality of trials and meta-analysis was performed. Seven trials were included, where six healthy subjects and one subject with poorer cognitive functions were recruited. One trial had unclear ROB while the rest had high ROB. For executive function tests, there was a significant difference favoring CE (pooled standardized mean difference (SMD) of −0.55 (−1.04, −0.06)) and another with no significant difference (pooled SMD of 0.70 (−0.001, 1.40)). For short-term memory tests, no significant difference was found (pooled SMD of 0.63 (−0.16, 1.42)). Currently, there is a lack of convincing evidence to show a cognitive enhancing effect of CE.

## 1. Introduction

Chicken essence (CE) is the cooked, concentrated, liquid extract from chicken, which is a popular traditional remedy amongst Asians [1,2,3,4,5,6]. In general, CE consists of major components of dipeptides and free amino acids, where carnosine and anserine are the most concentrated active ingredients [7,8]. Despite having a number of different CE companies in the market, the only variation found between commercial CE preparations is either with [9,10] or without [11,12] the addition of caramel as coloring agent. Combinational CE preparations are also commonly available, for example, with the addition of ginseng, cordyceps, or dong guai [10,11,12].

Over the past 20 years, CE company has claimed that the health benefits of CE are scientifically proven, with support from research conducted in Asia and the UK [9]. Promising results were reported in research in terms of improvements in cognitive functions [2], mental fatigue [13], and mood [5], although most of these studies were sponsored by CE companies. The marketing of CE focused on consumers intend to improve cognitive functions, as well as stress and fatigue, immunity, and general well-being [9,10,11,12].

In recent year, an animal study [7] found that oral administration of CE to rats increased the concentration of carnosine and anserine in the brain. However, there was no clear indication how this can affect cognitive functions in human beings. A literature review [1], funded by CE company, summarized all the general effects of CE. However, in the review, the authors did not critically appraise a specific effect of CE with a clear-cut research question [1].

A number of randomized controlled trials (RCTs) [2,3,4,5,13,14,15] have been conducted to investigate the effects of CE in cognitive function improvement. However, there is a lack of critical appraisal and summary of the effects of CE in improving cognitive functions. This systematic review aims to critically appraise and summarize all the available evidence to determine the effects of CE in improving cognitive functions as well as its safety.

## 2. Materials and Methods

This systematic review was performed in accordance with the Preferred Reporting Items for Systematic Review and Meta-analysis (PRISMA) [16].

### 2.1. Protocol and Registration

The review protocol is registered with PROSPERO (registration number: CRD42015023474).

### 2.2. Data Sources and Search Strategy

We electronically searched for relevant articles published from inception to 30 September 2015. We searched MEDLINE, EMBASE, CINAHL Plus, Cochrane Central Register of Controlled Trials (CENTRAL), AMED (The Allied and Complementary Medicine Database), KoreaMed as well as local Chinese and Thai databases. Keywords used were chicken essence, chicken extract or chicken meat ingredients, and brain, cognition, memory, attention, analysis, or mathematics. There was no language restriction. In addition, bibliographies of relevant articles were examined to identify potential studies not indexed in aforementioned databases. Authors of relevant articles were enquired if they were aware of other relevant published or unpublished studies.

### 2.3. Study Selection

Studies were included if they were RCTs involving human subjects. The RCTs included were those which used chicken essence (CE) in the intervention group compared to at least one comparator. Test to assess cognitive functions must be employed in the RCTs. Studies included must provide adequate information related to the cognitive effects of CE, and study characteristics for data extraction. In the context of this review, cognitive effect refers to any domain of memory, language, attention, executive function, and information processing speed [17]. Studies were screened by two independent reviewers (SLT and SS). Initially, title and abstract of articles were screened to identify potentially relevant studies. Thereafter, full-text of relevant studies were retrieved and reviewed.

### 2.4. Data Extraction

Characteristics and results of trials were extracted by two independent reviewers (SLT and SS) using a standardized data extraction sheet. Any disagreement was resolved by discussion. Study designs, blinding status, characteristics of subjects, chicken essence, comparator, cognitive function test, duration of intake, interval of assessment, and funding status were extracted.

The characteristics extracted for cognitive function tests were the name of the cognitive function test, cognitive function domain, outcome measure and scale employed. The cognitive function domain was determined by understanding the procedure of each test. Each test was categorized into one domain which is the primary domain, to prevent overweighing on one test and to enable consistent analysis throughout the review [18]. The outcome measurement of the cognitive function test was the primary outcome of interest. In addition, the adverse effects such as thirst and decreased bowel movements reported in the trials were the secondary outcome of interest. Data not available was requested directly by the corresponding author.

### 2.5. Study Quality Assessment

The methodological quality of each trial was assessed by two independent reviewers (SLT and SS) using a risk of bias (ROB) tool [19]. The domains for methodological evaluation using ROB tool include sequence generation, allocation concealment, blinding of participants, personnel and outcome assessors, incomplete outcome data, selective outcome reporting, and other sources of bias [19]. The funding of the trials was assessed in the domain of “other sources of bias”. Each trial was classified as having low risk (low risk of bias for all domains), high risk (high risk of bias for one or more domains), or unclear risk (unclear risk of bias for one or more key domains, given no high risk of bias in any domain). In addition, Jadad score was determined for each trial by assessing randomization, double-blinding, and the account of all subjects [20].

### 2.6. Data Analysis

To determine the cognitive effect of CE, data of individual cognitive function tests were compared between CE group and comparator group using mean difference (MD) with 95% confidence interval (CI). Cognitive function tests with the same name, domain, outcome measure, and scale were pooled using weighted mean difference (WMD). Cognitive function tests with the same domain, outcome measure and scale, albeit with different names, were pooled using standardised mean difference (SMD). Heterogeneity of the included trial cognitive function tests was assessed using chi-squared test and *I*^2^ test. Chi-squared test p-value of 0.10 or less indicates statistical significance of heterogeneity [19]. *I*^2^ value of more than 50% indicates substantial heterogeneity [19]. Data from trials were pooled in a meta-analysis using a random-effects model [21] with 95% CI. The software used for data analysis was STATA^®^ version 12 (STATA Corp, College Station, TX, USA).

### 2.7. Quality of Evidence

The overall quality of evidence was assessed using Grading of Recommendations Assessment, Development, and Evaluation (GRADE) approach [22] that considered study design, ROB of individual trials, heterogeneity, directness of evidence, precision of effect estimates, and possibility of publication bias [22]. GRADEpro^®^ version 3.6.1 (McMaster University, Hamilton, ON, Canada, 2014) software was used to generate the summary of findings (SoF) table. The overall quality of evidence ranged from high, moderate, low, to very low quality where high quality indicates the estimated effect lies close to the true effect while very low quality indicates the estimated effect is likely to be substantially different from the true effect [22].

### 2.8. Sensitivity Analysis

To ensure robustness of results, sensitivity analysis was performed by (1) meta-analysis using a fixed-effect model when there is no heterogeneity [23]; and (2) excluding data of trials with low quality from the meta-analysis.

## 3. Results

### 3.1. Study Selection

**Figure 1:**
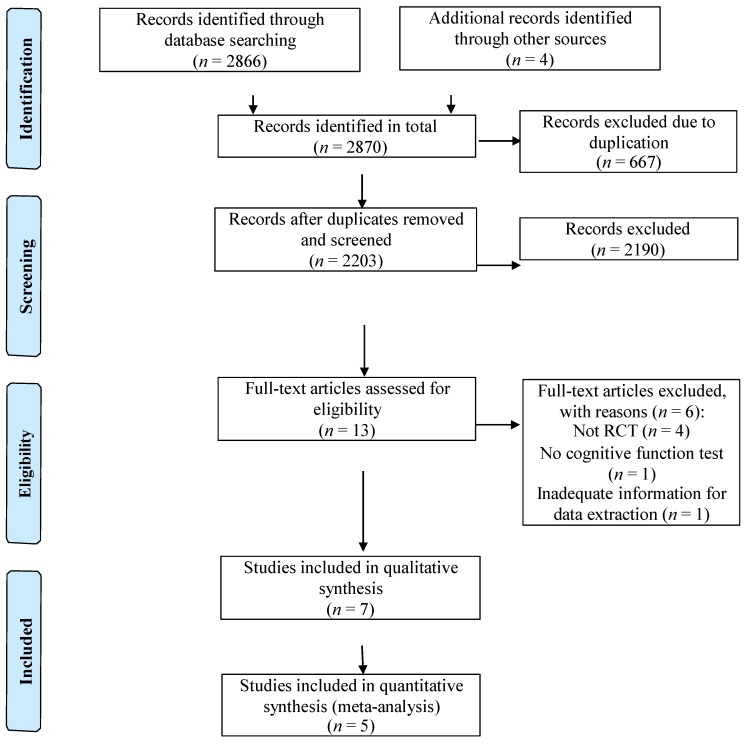
Flow of study selection.

Our search yielded a total of 2870 potential articles including 2866 from electronic databases, two from bibliographies of relevant articles, and two from contacting authors. Six hundred and sixty-seven duplicates were removed. Of the remaining 2203 studies screened, only 13 were relevant and were retrieved to be reviewed in full-text. During the full-text screening, only seven studies met the inclusion criteria. The six excluded studies either were non-RCT (*n* = 4), did not employ a cognitive function test (*n* = 1), and had inadequate information for data extraction (*n* = 1). As a result, a total of seven trials involving a total of 363 participants were included in this review. The trials were conducted in Malaysia [2,14,15], Japan [3,4,13], and United Kingdom [5]. The flow of study selection was shown in Figure 1.

### 3.2. Study Characteristics

The study characteristics were summarized in Table 1. Out of the seven trials, four [2,5,14,15] employed parallel design, and three [3,4,13] employed cross-over design. Out of the three trials, two [4,13] employed paired-analysis and only one [4] mentioned that a within-subject comparison was performed. The trial [3] which employed unpaired analysis did not mention whether the first or second period of cross-over trial was used for analysis. The study designs of the cross-over trials were summarized in Table 2.

**Table 1:** Study characteristics of included trials.

Author	RCT Design	Blinding Status (Stated by Author)	No. of Participants (Chicken Essence Group) ITT; Completed	No. of Participants (Placebo Group) ITT; Completed	Participants’ Condition	Participants’ Age Mean (Range)	Chicken Essence and Placebo’s Form	Placebo	Test to Check Placebo’s Similarity	Quantity	Intake Duration (Days)	Interval Assessed (Days)	Risk of Bias; Jadad Score
Nagai 1996, [4]	Cross-over	-	Total = 20; 16 #		Healthy volunteer	21.1 (18–24)	Liquid ~	Gelatin, caramel	-	140 mL	7	0,7	High; 1
Azhar 2003, [14]	Parallel	Double-blinded	60; 56	57; 52	Healthy volunteer §	−(23–24)	Liquid	Water, caramel	-	70 mL	14	0, 14	High; 1
Azhar 2008, [15]	Parallel	Double-blinded	Total ITT= 102 `; 38; 31		Healthy volunteer §	23 (22–24)	Liquid	Milk protein	-	70 mL	14	0, 14	High; 1
Azhar 2013, [2] ^	Parallel	Double-blinded	Total ITT= 46 `; 10; 10		Walk-in or general practitioner referred patients with poorer cognitive function	47.5 (35–65)	Tablet *	Microcrystalline cellulose	-	670 mg	42	0, 42, 56	High; 2
Konagai 2013, [3]	Cross-over	Double-blinded	Total = 12; 12 #		Healthy, elderly volunteer	62.3 (60–68)	Liquid *	Milk casein, caramel	-	140 mL	7	0,7	High; 1
Yamano 2013, [13] ^	Cross-over	-	Total = 20; 20 #		Healthy volunteer, male	34.7 (33–35)	Liquid *	Milk casein, caramel	-	140 mL	28	0, 7, 28	High; 1
Yamano 2015, [5] ^	Parallel	Double-blinded	24; 24	22; 22	Healthy volunteer	21.5–22.2 (-)	Liquid *	Milk casein, caramel	Yes	70 mL	10	0, 10	Unclear; 3

RCT, Randomized controlled trial; ITT, Intention-to-treat; - Not reported; # Participants in each group not reported; ~Brands chicken essence (Cerebos Pacific Limited, Singapore) was used; § Subjects were considered as generally healthy subjects in this review, although they were claimed as stressed in the trials; ` Intention-to-treat sample size only reported as a whole and not in separate groups; ^ Study funded by Cerebos Pacific Limited, Singapore; * Both chicken essence and placebo were supplied by Cerebos Pacific Limited, Singapore.

**Table 2:** Study design of cross-over trials.

Author	Paired/Unpaired Analysis	For Paired Analysis, within-Subject Comparison Performed?	For Unpaired Analysis, First or Second Period Used?
Nagai 1996, [4]	Paired	Yes	Not applicable
Konagai 2013, [3]	Unpaired	Not applicable	Not reported
Yamano 2013, [13]	Paired	Not reported	Not applicable

The total intention-to-treat number of subjects in each trial was relatively small, ranged from 20 [4] to 117 [14]. Subjects in the included trials were in the age-ranges of 18–24 [4,5,14,15], 33–35 [13], 35–65 [2], and 60–68 [3]. None of the trials assessed the effect of CE in children. Subjects in almost all (6/7) trials [3,4,5,13,14,15] were generally healthy. Although two trials [14,15] claimed that the subjects were in stressed condition, without stress level assessed, they were considered as generally healthy in this review. For one trial [2], the subjects were walk-in or general practitioner referred patients with poorer cognitive function.

All the included trials used placebo as the comparator. One trial [14] also used carrageenan as a comparator in addition to placebo. Almost all (6/7) trials [3,4,5,13,14,15] used liquid form of CE and comparators, while one trial [2] used tablet form. CE and placebo were supplied by a CE company (Cerebos Pacific Limited, Singapore) in four trials [2,3,5,13]. One trial [4] used Brand’s^®^ CE of Cerebos Pacific Limited, Singapore. Two trials [14,15] did not mention the source of the CE and comparators. The ingredients used for placebo in liquid form were mixture of milk casein and caramel [3,5,13], milk casein [15], water and caramel [14], and gelatin and caramel [4]. Placebo in tablet form of one trial [2] was made up of microcrystalline cellulose.

The quantity of CE and comparator in liquid administered for six trials were a daily dose of 70 mL [5,14,15] and 140 mL [3,4,13]. Three trials [3,5,13] quantified the active ingredients of CE, all of which were sourced from literature. CE in tablet of one trial [2] was administered with a daily dose of 670 mg, where the active ingredient was termed as “chicken meat ingredient-168”. The duration of intake varied from the range of seven days [3,4] the shortest to forty-two days [2] the longest. In addition to the assessment of cognitive functions after the last day of CE intake, one trial [2] also assessed the delayed-effect two weeks after discontinuing CE.

### 3.3. Study Quality Assessment

With assessment of quality using ROB tool, almost all (6/7) trials [2,3,4,13,14,15] were found to have overall high ROB. One trial [5] was found to have overall unclear ROB. ROB assessment for each domain was summarized in Table 3.

**Table 3:** Assessment of quality of trials using risk of bias (ROB) tool.

	Sequence Generation	Allocation Concealment	Blinding	Incomplete Outcome Data	Selective Reporting	Other Sources of Bias	Overall
Nagai 1996, [4]	High	Unclear	High	Low	Low	Unclear	High
Azhar 2003, [14]	Unclear	Unclear	High	Low	Low	Unclear	High
Azhar 2008, [15]	Unclear	Unclear	High	High	Low	Unclear	High
Azhar 2013, [2]	Unclear	Unclear	Unclear	High	High	High	High
Konagai 2013, [3]	Unclear	Unclear	High	Unclear	Low	High	High
Yamano 2013, [13]	Unclear	Unclear	High	Unclear	Low	Unclear	High
Yamano 2015, [5]	Unclear	Unclear	Unclear	Low	Low	Low	Unclear

Almost all (6/7) trials [2,3,5,13,14,15] were found to have unclear ROB for “sequence generation” domain. Even though “randomized” was mentioned in the six trials [2,3,5,13,14,15], there was no description of methods of sequence generation. One trial [4] was found to have high ROB for “sequence generation” domain as the approach employed was non-randomized, where the allocation was determined by the result of a pre-arithmetic calculation test. All trials did not describe any component of “allocation concealment”.

For “blinding” domain, five trials [3,4,13,14,15] were found to have high ROB while two trials [2,5] had unclear ROB. The high ROB of blinding was because the liquid placebo used in the trials [3,4,5,13,14,15] could possibly have a different smell and taste from CE. In fact, taste difference between placebo and CE were documented in the trials [14,15]. Only one trial [5] conducted a test to check blinding of subjects.

For “incomplete outcome data” domain, three trials [4,5,14] were found to have low ROB as all withdrawal and dropouts were adequately reported with sound reasons, while two trials [3,13] were found to have unclear ROB as there was no description of withdrawal or dropouts. For the remaining two trials [2,15] with high ROB, one [15] had excluded around one-third (33/102) of the initial sample size for the final data synthesis in addition to incomplete reporting of (8/102) missing data, while another [2] had excluded more than half of the initial sample size (26/40). As for the “selective reporting” domain, one trial [2] was found to have high ROB as there was one pre-specified test outcome which was not reported. The remaining trials [3,4,5,13,14,15] were found to have low ROB for this domain.

For “other sources of bias” domain, three trials [2,5,13] clearly reported that they received funding from a CE company (Cerebos Pacific Limited, Singapore) while four trials [3,4,14,15] did not mention their source of funding. Of the three trials funded by the CE company, only one trial [5] was found to have low ROB as there was a declaration of no conflict of interest as the funding company was not involved in any part of the trial or manuscript preparation, besides the supply of CE and placebo. The other two trials [2,13] were found to have high ROB as the authors were affiliated with CE company and involved in trial design and conduct, data analysis, and manuscript preparation. The detailed assessment of ROB was summarized in Appendix A.

Jadad score was one for five trials [3,4,13,14,15], two for one trial [2], and three for one trial [5]. The assessment using Jadad scale was summarized in Table B1.

### 3.4. Effects of Chicken Essence in Cognitive Function Improvement

Of the seven included trials, 36 cognitive function outcome measurements were found where 30 had complete data available for data analysis. To ensure consistent comparison throughout the review, (1) data of carrageenan (comparator) group [14] and (2) data of delayed effect of CE [2] were not included in the analysis. Of the 30 complete data, 13 were found to have significant difference between CE group and placebo group, where eight were in executive function domain, and five in short-term memory domain. The detailed results of individual test were summarized in Table 4.

**Figure C1:**
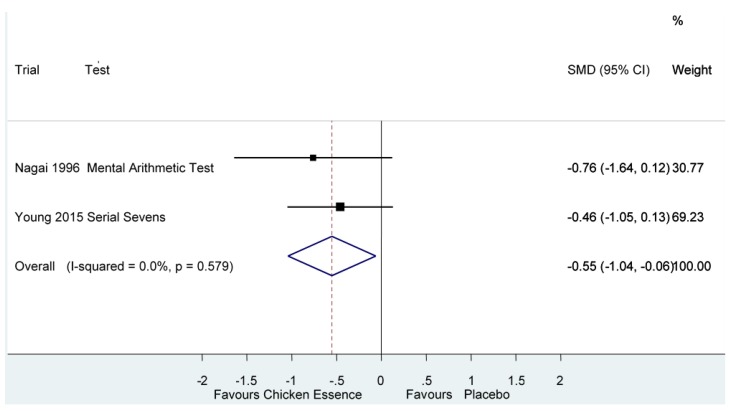
Pooled effect estimate of score of error and error rate of executive function tests. SMD, Standardized mean difference; CI, Confidence interval.

**Figure C2:**
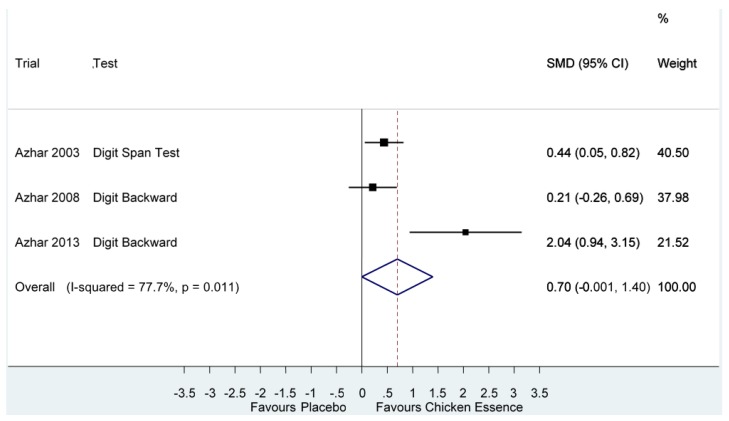
Pooled effect estimate of score of longest list of digits remembered of executive function tests.

Outcome measures of the cognitive function tests employed in the seven included trials were oxy-hemoglobin concentration [3], score with scales of error [5], error rate [4], longest list of digits or/and words remembered [2,14,15], or with scale not reported [2,14,15], reaction time of decision and movement [5] or with scale not reported [5,13]. Data of oxy-hemoglobin concentration [3] was not pooled with others because we did not consider oxy-hemoglobin concentration a direct and validated measurement of cognitive function. Data of reaction time was not reported from one trial [13]. Consequently, five trials [2,4,5,14,15] were included for meta-analysis. Three pooled results were obtained from the meta-analysis, where two were in the executive function domain, and one in the short-term memory domain. Pooled results were summarized in Table 5. Forest plots of the three pooled results can be found in Figure C1, Figure C2 and FigureC3.

**Table 4:** Characteristics of cognitive function tests.

Cognitive Function Domain	Cognitive Function Test	Study	Outcome Measure (Scale)	Additional Information of Outcome Measure/Scale	Range of Score Reported	Mean Difference (95% CI)
Attention	Simple Reaction Task	Konagai 2013, [3]	Oxy-hemoglobin concentration (NA)	The presence of oxy-hemoglobin in brain during cognitive function test	NR	NR
	EE-Arrow Flankers Test (Congruent)	Yamano 2015, [5]	Score (Error) $	Number of incorrect answer of cognitive function test	−2.8–1.3	−0.33 (−0.91, 0.26)
	EE-Arrow Flankers Test (Neutral)	Yamano 2015, [5]	Score (Error) $	Number of incorrect answer of cognitive function test	−2.8–1.3	−0.24 (−0.82, 0.34)
	EE-Arrow Flankers Test (Incongruent)	Yamano 2015, [5]	Score (Error) $	Number of incorrect answer of cognitive function test	−2.8–1.3	−0.47 (−1.06, 0.11)
	EE-Arrow Flankers Test (Congruent)	Yamano 2015, [5]	Reaction time (NR ~) $	Time used to complete cognitive function test	470.9–1184.6	−0.001 (−0.58,0.58)
	EE-Arrow Flankers Test (Neutral)	Yamano 2015, [5]	Reaction time (NR ~) $	Time used to complete cognitive function test	470.9–1184.6	−0.10 (−0.68, 0.48)
	EE-Arrow Flankers Test (Incongruent)	Yamano 2015, [5]	Reaction time (NR ~) $	Time used to complete cognitive function test	470.9–1184.6	0.13 (−0.45, 0.71)
	Jensen-Simple and Choice Reaction Time	Yamano 2015, [5]	Reaction time (Decision time ~) $	Time used to complete cognitive function test	122.4–671.1	−0.36 (−0.92, 0.24)
	Jensen-Simple and Choice Reaction Time	Yamano 2015, [5]	Reaction time (Movement time ~) $	Time used to complete cognitive function test	132.1–661.8	−0.51 (−1.10, 0.08)
Executive Function	Nagai’s Mental Arithmetic Test	Nagai 1996, [4]	Score (Error rate) $	Percentage of incorrect answer out of the filled answer	NR	−0.76 (−1.64, 0.12)
	The Three Minute Memory Test	Azhar 2003, [14]	Score (NR ~) $	NR	1.1–8.7	0.16 (−0.22, 0.54)
	WAIS-Digit Span	Azhar 2003, [14]	Score (Longest list ~) $	Longest list of digits remembered	11.3–32.7	0.23 (−0.15, 0.61)
	Mental Arithmetic Test	Azhar 2003, [14]	Score (NR ~) $	NR	2.8–14.7	0.98 (0.34, 1.62) *
	WAIS-Arithmetic Test	Azhar 2008, [15]	Score (NR ~) $	NR	9.6–26.8	0.73 (−1.27, 2.73) *
	WAIS-Digit Backward	Azhar 2008, [15]	Score (Longest list ~) $	Longest list of digits remembered	5.2–10.7	0.47 (−0.57, 1.51)
	WAIS-Digit Backward	Azhar 2013, [2]	Score (Longest list ~) $	Longest list of digits remembered	24.2–28.3	2.00 (1.14, 2.86) *
	Letter Number Sequencing	Azhar 2008, [15]	Score (Longest list ~) $	Longest list of letters and digits remembered	8.9–16.1	0.31 (−0.17, 0.78)
	Letter Number Sequencing	Azhar 2013, [2]	Score (Longest list ~) $	Longest list of letters and digits remembered	17.2–19.6	6.25 (4.03, 8.47) *
	RAVLT-Delayed Recall	Azhar 2013, [2]	Score (Longest list ~) $	Longest list of words remembered	17.7–6.4	3.33 (1.94, 4.73) *
	RAVLT-Recall	Azhar 2013, [2]	Score (Longest list ~) $	Longest list of words remembered	6.6–17.7	2.81 (1.54, 4.08) *
	RAVLT-Retroactive Interference	Azhar 2013, [2]	Score (Longest list ~) $	Longest list of words remembered	5.9–19.2	4.06 (2.47, 5.65) *
	Working Memory Test	Konagai 2013, [3]	Oxy-Hemoglobin Concentration (NA) $	The presence of oxy-hemoglobin in brain during cognitive function test	NR	0.20 (0.06, 0.34) *
	Traffic Light’s Test	Yamano 2013, [13]	Reaction time (NR ~)	Time used to complete cognitive function test	NR	NR
	Stroop Test	Yamano 2013, [13]	Reaction time (NR ~)	Time used to complete cognitive function test	NR	NR
	Serial Sevens	Yamano 2015, [5]	Score (Error) $	Number of incorrect answer of cognitive function test, time used to complete cognitive function test	−3.8	−0.46 (−1.05, 0.13)
	Serial Sevens	Yamano 2015, [5]	Reaction time (NR ~) $	Time used to complete cognitive function test	705.7–3032.9	0.32 (−0.27, 0.90)
Long-term Memory	Non-Stroop Test	Yamano 2013, [13]	Reaction time (NR ~)	Time used to complete cognitive function test	NR	NR
Short-term Memory	Short-term Memory Test	Nagai 1996 [4]	Score (Error rate)	Percentage of incorrect answer out of the filled answer	NR	NR
	Mental Comprehension Test	Azhar 2003, [14]	Score (NR ~) $	NR	0.6–11.4	0.64 (0.25, 1.02) *
	WAIS-Digit Forward	Azhar 2008, [15]	Score (NR ~) $	Longest list of digits remembered	8.4–20.7	−0.10 (−0.57, 0.37)
	RAVLT-Proactive Interference	Azhar 2013, [2]	Score (NR ~) $	Longest list of words remembered	7.5–19.1	3.75 (2.24, 5.26) *
	RAVLT-Immediate Memory	Azhar 2013, [2]	Score (NR ~) $	NR	8.0–20.4	3.92 (2.37, 5.47) *
	RAVLT-Best Learning	Azhar 2013, [2]	Score (NR ~) $	NR	8.5–21.4	1.70 (0.66, 2.74) *
	RAVLT-Total Learning	Azhar 2013, [2]	Score (NR ~) $	NR	43.8–105.6	6.50 (2.66, 10.34) *
Visuospatial Skills	Figures Construction Test	Azhar 2003, [14]	Score (NR ~) $	NR	1.2–7.9	0.28 (−0.10, 0.66)
	Milner and Snyder-Groton Maze Learning Test	Konagai 2013, [3]	Oxy-hemoglobin concentration (NA)	The presence of oxy-hemoglobin in brain during cognitive function test	NR	NR
(Subdomain) Information Processing Speed	Traffic Light’s Test	Yamano 2013, [13]	Reaction time (NR ~)	Time used to complete cognitive function test	NR	NR
	Stroop Test	Yamano 2013, [13]	Reaction time (NR ~)	Time used to complete cognitive function test	NR	NR
	Non-Stroop Test	Yamano 2013, [13]	Reaction time (NR ~)	Time used to complete cognitive function test	NR	NR
	EE-Arrow Flankers Test (Congruent)	Yamano 2015, [5]	Reaction time (NR ~)	Time used to complete cognitive function test	470.9–1184.6	−0.00 (−0.58,0.58)
	EE-Arrow Flankers Test (Neutral)	Yamano 2015, [5]	Reaction time (NR ~)	Time used to complete cognitive function test	470.9–1184.6	−0.10 (−0.68, 0.48)
	EE-Arrow Flankers Test (Incongruent)	Yamano 2015, [5]	Reaction time (NR ~)	Time used to complete cognitive function test	470.9–1184.6	0.13 (−0.45, 0.71)
	Jensen-Simple and Choice Reaction Time	Yamano 2015, [5]	Reaction time (Decision time ~) $	Time used to complete cognitive function test	122.4–671.1	−0.36 (−0.92, 0.24)
	Jensen-Simple and Choice Reaction Time	Yamano 2015, [5]	Reaction time (Movement time ~) $	Time used to complete cognitive function test	132.1–661.8	−0.51 (−1.10, 0.08)
	Serial Sevens	Yamano 2015, [5]	Reaction time (NR ~)	Time used to complete cognitive function test	705.7–3032.9	0.32 (−0.27, 0.90)

* Result with significant difference; NA, Not applicable; NR, Not reported; EE, Eriksen and Eriksen; $ Result with complete data for analysis; ~ Result that followed the trend that higher score or shorter reaction time indicates better cognitive functions; WAIS, Wechsler Adult Intelligence Scale; RAVLT, Rey Auditory Verbal Learning Test.

**Table 5:** Pooled results of meta-analysis using a random-effects model.

Cognitive Area	Study	ROB	Test	Outcome Measure	Pooled SMD (95% CI)
Executive Functions	Nagai 1996, [4]	High	Nagai’s Mental Arithmetic Test	Score (Error rate)	−0.55 (−1.04, −0.06) * *I*^2^ = 0%, *p* = 0.58
	Yamano 2015, [5]	Unclear	Serial Sevens	Score (Error)	
	Azhar 2003, [14]	High	Digit Span Test	Score (Longest list)	0.70 (−0.001, 1.40) *I*^2^ =77.7%, *p* = 0.01
	Azhar 2008, [15]	High	Digit Backward	Score (Longest list)	
	Azhar 2013, [2]	High	Digit Backward	Score (Longest list)	
Short-term Memory	Azhar 2003, [14]	High	Mental Comprehension Test	Score (NR ~)	0.63 (−0.16, 1.42) *I*^2^ = 82.9%, *p* = 0.00
	Azhar 2008, [15]	High	Digit Forward	Score (NR ~)	
	Azhar 2013, [2]	High	Best Learningβ	Score (NR ~)	

ROB, Risk of bias; SMD, Standardized mean difference; CI, Confidence interval; * Result with significant difference; NR, Not reported; ~ Results that followed the trend that higher score or shorter reaction time indicates better cognitive functions; βBest Learning test was chosen from Azhar 2013, [2] because the range of reported score was the closest to the range of score of two other tests pooled together (as we did not know which scale was employed), and its standard deviation is the widest (*i.e.*, conservative estimate).

For executive function domain, we had included five trials [2,4,5,14,15] with 259 subjects in two separate analyses. For the first analysis, a significant pooled SMD of −0.55 (95% CI: −1.04, −0.06) with no heterogeneity (*I*^2^ = 0%, *p* = 0.58) was observed among two included trials [4,5] with 62 subjects. The score of error rate and error for executive function tests were pooled from two trials with high [4] and unclear ROB [5]. The scores were pooled, albeit with different scales, because they followed the trend where lower score indicates better cognitive functions. The data of the cross-over trial [4] were pooled with another trial [5] which was parallel [19].

**Figure D1:**
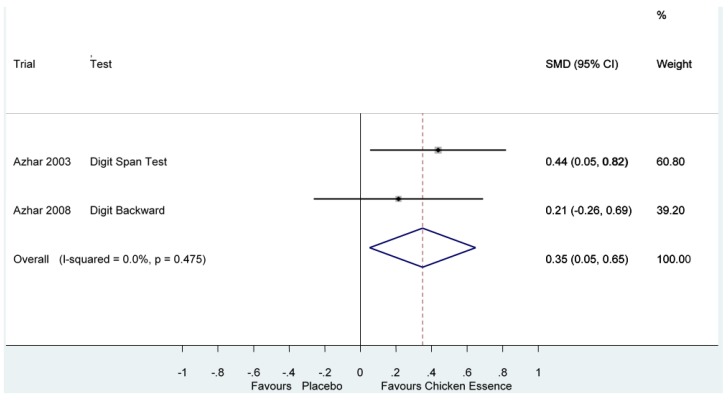
Pooled effect estimate of score of longest list of digits remembered of executive function tests (with Azhar’s test removed [2]).

For the second analysis of the executive function domain, a non-significant pooled SMD of 0.70 (95% CI: −0.001, 1.40) with substantial heterogeneity (*I*^2^ = 77.7%, *p* = 0.01) was obtained from the score with scale of longest list of digits of the three trials [2,14,15] with high ROB which involved 197 subjects. The substantial heterogeneity can possibly be explained by the difference of subjects in the trials. The sample in one of the trials [2] was older with an age-range of 35–65, and with poorer cognitive functions, whereas the subjects of the other two trials [14,15] were of the age-range of 22–24 and generally healthy. In addition, the regimens of CE administered were different where one trial [2] used CE in tablet form for a more prolonged period of 42 days compared to the two trials [14,15] which used CE in liquid form for a period of 14 days. When the trial [2] with different subject characteristics and CE regimen was removed, a significant pooled SMD of 0.35 (0.05, 0.65) with no heterogeneity (*I*^2^ = 0%, *p* = 0.475) was obtained; pooled SMD was not changed when fixed-effect model was used. The forest plot of the pooled result can be found in Figure D1.

**Figure D2:**
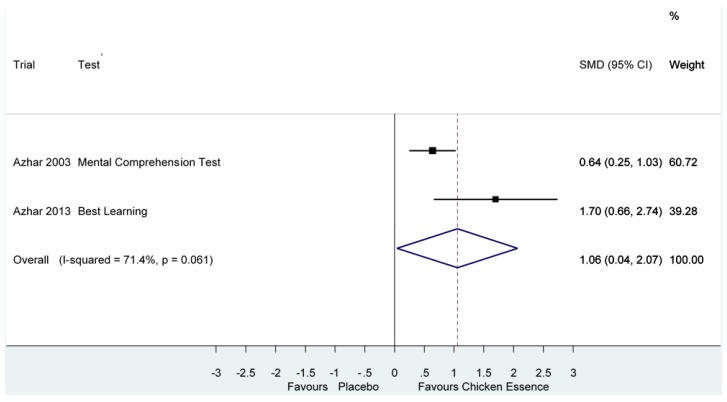
Pooled effect estimate of score of short-term memory tests (with Azhar’s test removed [15]).

**Figure D3:**
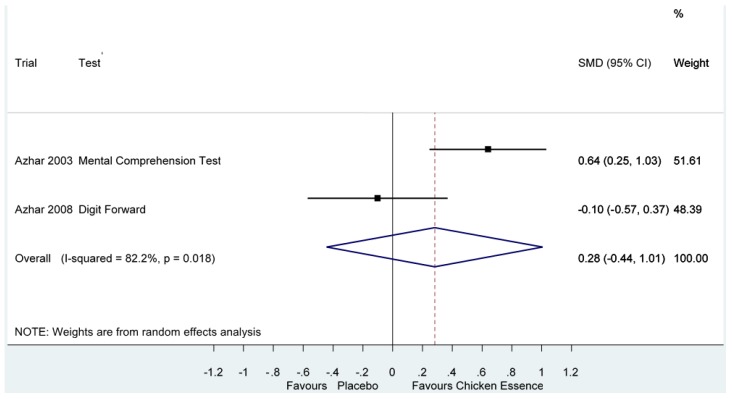
Pooled effect estimate of score of short-term memory tests (with Azhar’s test removed [2]).

**Figure D4:**
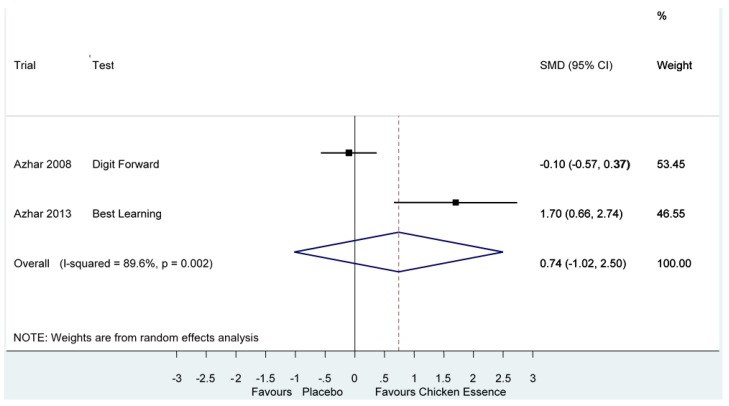
Pooled effect estimate of score of short-term memory tests (with Azhar’s test removed [14]).

For short-term memory domain, a non-significant pooled SMD of 0.63 (−0.160, 1.42) with substantial heterogeneity (*I*^2^ = 82.9%, *p* = 0.001) was obtained from the scores of three trials [2,14,15] with high ROB which involved 197 subjects. Although the scales employed in the three trials were not reported, the data were pooled as they followed the trend that higher score indicates better cognitive functions. We explored the causes of heterogeneity and could not identify the clear source. Further meta-analysis was performed by removing either one of three trials from the pooled results, all of which still produced substantial heterogeneity. The forest plots of the pooled result can be found in Figure D2, Figure D3 and Figure D4.

### 3.5. Adverse Effects

Adverse effects of consuming CE were assessed in two out of seven trials [2,5]. One trial [5] reported no significant difference between CE group and placebo group on the reported rates of thirst, decreased bowel movements, tiredness, and slight insomnia. No adverse effect was recorded in another trial [2].

### 3.6. Quality of Evidence

Using GRADE approach, the quality of evidence to recommend CE for improving executive functions was found to be either low or very low. The same scenario applied for recommendation of short-term memory where the quality of evidence was very low. The low quality of evidence was due to the reasons that (1) individual studies had low quality (high and unclear ROB); and (2) inconsistency of effect in cognitive functions improvement with substantial heterogeneity. Summary of findings (SoF) table was presented in Table 6.

### 3.7. Sensitivity Analysis

It was not possible to perform sensitivity analysis by excluding data of trials with low quality from the meta-analysis due to the reasons that (1) a limited number of trials were pooled and (2) all trials were of low quality (high or unclear ROB).

**Table 6:** Summary of findings (SoF) table for cognitive function outcomes of meta-analysis measured in clinical trials of chicken essence. §

Outcomes	Relative Effect (95% CI)	No of Participants (Studies)	Quality of the Evidence (GRADE)
**Executive Functions** Error Rate Follow-up: 7–10 days	The mean executive functions in the intervention groups was **0.55 standard deviations lower** (1.04 to 0.06 lower)	62 (2 studies)	⊕⊕⊝⊝ **low** ^1^
**Executive Functions** Performance Score Follow-up: 2–8 weeks	The mean executive functions in the intervention groups was **0.70 standard deviations higher** (0.001 lower to 1.4 higher)	197 (3 studies)	⊕⊝⊝⊝ **very low** ^2,3^
**Short-term Memory** Performance Score Follow-up: 2–8 weeks	The mean short-term memory in the intervention groups was **0.63 standard deviations higher** (0.16 lower to 1.42 higher)	197 (3 studies)	⊕⊝⊝⊝ **very low** ^2,4^

§ Among studies that compared chicken essence with placebo in healthy subjects and subjects with poorer cognitive functions. ^1^ Both studies have unclear risk of bias; ^2^ All 3 studies have high risk of bias; ^3^ Very high *I*^2^ value of 77.7%; ^4^ Very high *I*^2^ value of 82.9%. CI: Confidence interval. GRADE Working Group grades of evidence—**High quality:** Further research is very unlikely to change our confidence in the estimate of effect; **Moderate quality:** Further research is likely to have an important impact on our confidence in the estimate of effect and may change the estimate; **Low quality:** Further research is very likely to have an important impact on our confidence in the estimate of effect and is likely to change the estimate; **Very low quality:** We are very uncertain about the estimate.

## 4. Discussion

To the extent of our knowledge, this is the first systematic review which critically appraises and summarizes the available evidence about the effects of CE in improving cognitive functions. Based on a combination of cognitive function tests, the pooled effects were significant for some measures (in the executive function domain) and not significant for other measures (in the executive function and short-term memory domains). There were great uncertainties on the possible effect sizes, although it appeared that the effects, if present, were at best modest and non-clinically significant. Therefore, the overall findings need to be interpreted with caution.

The findings of our review was consistent with the RCTs [24,25] evaluating the effects of carnosine (the active ingredient of CE). The two RCTS which also employed a combination of cognitive function tests, in schizophrenia patients [24] and Persian Gulf War veterans [25], respectively, found that the effects were only significant for certain cognitive function tests but not all. The imminently inconclusive findings about the effect of cognitive properties of CE can be related to the methodological issues of cognitive function tests. Future RCTs intended to employ cognitive function tests should consider referring to guidance available [17,26] in order to make a sound selection of appropriate cognitive function tests.

A study report [26] proposed that the change of attention can contribute to relative change to other cognitive function domains. In our review, however, given the limited number of trials included, there was no summary of effect given for attention domain. With the findings of individual attention tests, all the results were insignificant. This could imply that any effect of CE on the executive function domain might not be due to the change of attention based on our review. However, more future RCTs should employ attention tests concurrently with tests assessing other cognitive function domains in order to justify this relationship.

As with any systematic review and meta-analysis, our review shares the limitations of the original trials. Firstly, the quality of included trials was low with high or unclear ROB. Secondly, cognitive function tests employed in the trials provided an ambiguous interpretation of cognitive-enhancing effects of CE. However, based on the most reasonably applicable methods employed to generate quantitative synthesis (meta-analysis), in addition to qualitative synthesis, this review provided important information about the direction of the cognitive-enhancing effects of CE and the designs and characteristics of the RCTs evaluating the cognitive functions of CE conducted so far. Lastly, although we have performed an exhaustive literature search, there might be unpublished study that we were unaware of. Due to the small number of trials included, publication bias was not tested. In a nutshell, the overall low and very low quality evidence based on GRADE indicates that a cautious interpretation of findings is warranted.

## 5. Conclusions

With the current evidence available, it is premature to support the claim that CE has cognitive-enhancing effects. More high quality RCTs are needed to better determine its effect.

Our review can potentially be used as information for healthcare-providers and the public to understand the current limited evidence of CE in supporting the claim of cognitive function improvement. Caution should be taken when interpreting health claims used for advertising nutritional products, especially in countries with a lack of a unified approach in the regulatory framework for health claims [27].

## Figures and Tables

**Figure C3:**
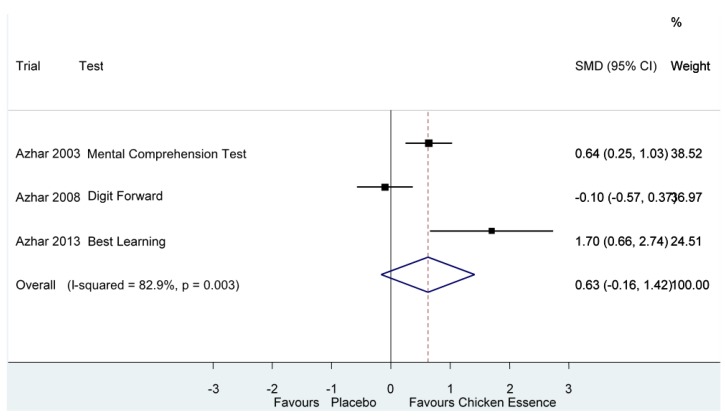
Pooled effect estimate of score of short-term memory tests.

## Appendix A. Assessment of quality of trials using Risk of Bias (ROB) tool.

**Table A1:** Risk of bias assessment of Nagai 1996 [4].

Items	Judgement	Specifics
Sequence Generation	High	Quote: “according to a pre-arithmetic calculation test to equalize in both groups the abilities to perform tasks“. Comment: A non-random approach was employed. Review authors believe this can introduce bias.
Allocation Concealment	Unclear	There was no description of allocation concealment.
Blinding	High	Quote: “same appearance and caloric content” Comment: The smell and taste of the placebo can differ from chicken essence (CE). Review authors believe this can introduce bias.
Incomplete Outcome Data	Low	Quote: “4/20 dropouts. (refused to continue the test)” Comment: Review authors believe no bias was introduced.
Selective Reporting	Low	Review authors believe no bias was introduced as all the pre-specified outcomes were adequately reported.
Other sources of bias	Unclear	There was no mention of funding, or declaration of conflict of interest.
**Overall**	High	

**Table A2:** Risk of bias assessment of Azhar 2003, [14].

Items	Judgement	Specifics
Sequence Generation	Unclear	Quote: “subjects were randomly divided”. Comment: Method of sequence generation was not specified.
Allocation Concealment	Unclear	There was no description of allocation concealment.
Blinding	High	Quote: “double-blind”; “taste difference between test samples”; “all subjects had not previously taken CEC”; “Investigator 1 who conducted the tests were blinded to the samples”; “Investigator 2 kept a record of all the samples”. Comment: The smell and taste of the placebo can differ from CE. Review authors believe this can introduce bias.
Incomplete Outcome Data	Low	Quote: “5/57 dropouts in placebo group, 4/60 dropouts in CE group, 3/58 dropouts in carrageenan group (due to personal reasons, non-compliance); 1/176 missing data”. Comment: Review authors believe no bias was introduced.
Selective Reporting	Low	Review authors believe no bias was introduced as all the pre-specified outcomes were adequately reported.
Other sources of bias	Unclear	There was no mention of funding, or declaration of conflict of interest.
**Overall**	High	

**Table A3:** Risk of bias assessment of Azhar 2008, [15].

Items	Judgement	Specifics
Sequence Generation	Unclear	Quote: “subjects were randomly divided”. Comment: Method of sequence generation was not specified.
Allocation Concealment	Unclear	There was no description of allocation concealment.
Blinding	High	Quote: “double-blind”; “placebo made up of milk protein (casein), as a comparison test sample”; “potential taste differences”; “most subject had not taken CEC previously, those who had taken it more than 10 years ago indicated they have no significant recollections”; “Investigator 1 who conducted the tests were blinded to the samples”; “Investigator 2 maintained a record of all the samples”. Comment: The smell and taste of the placebo can differ from CE. Review authors believe this can introduce bias.
Incomplete Outcome Data	High	Quote: “25/102 excluded (due to technical errors); 8/102 missing data”. Comment: One-third of the initial sample size (33/102) was excluded. Review authors believe this can introduce bias.
Selective Reporting	Low	Review authors believe no bias was introduced as all the pre-specified outcomes were adequately reported.
Other sources of bias	Unclear	There was no mention of funding, or declaration of conflict of interest.
**Overall**	High	

**Table A4:** Risk of bias assessment of Azhar 2013, [2].

Items	Judgement	Specifics
Sequence Generation	Unclear	Quote: “subjects were randomly divided”. Comment: Method of sequence generation was not specified.
Allocation Concealment	Unclear	Quote: “investigator did not know their group allocation” Comment: Method of allocation concealment was not specified.
Blinding	Unclear	Quote: “double-blind”; “placebo tablets contain microcrystalline cellulose”; “Investigator who conducted the tests were blinded to the information about the group allocation and samples provided”; “Independent investigator who has no information about the assessment maintained the record of all the samples and group allocation”. Comment: It is unclear whether the placebo had similar appearance and taste as CE tablet. Hence it is unclear whether if the study design was single- or double-blinded.
Incomplete Outcome Data	High	Quote: 26/40 excluded from data analysis due to either non-compliance of supplementation or withdrawal from the study. Comment: More than half of the initial sample size (26/40) was excluded. Review authors believe this can introduce bias.
Selective Reporting	High	One of the outcomes (DS Forward test) as mentioned in protocol was not reported. Review authors believe this can introduce bias.
Other sources of bias	High	The trial clearly mentioned that it was funded by CE company. In addition, it was clearly mentioned that three of the authors were affiliated with CE company where they were involved with study conduct, data analysis, and preparation of the manuscript. Review author believes this can introduce bias.
**Overall**	High	

**Table A5:** Risk of bias assessment of Konagai 2013, [3].

Items	Judgement	Description
Sequence Generation	Unclear	Quote: “volunteers were divided randomly”. Comment: Method of sequence generation was not specified.
Allocation Concealment	Unclear	There was no description on allocation concealment.
Blinding	High	Quote: “double-blind”; “placebo contained milk casein, caramel, and flavoring to yield proteins, calories, and color similar to CE”; “no subjects had previously taken CE”. Comment: Even placebo was reported to be prepared in a way to have proteins, calories, and color similar to CEC, placebo can still have different taste and smell compared to CE. Review authors believe this can introduce bias.
Incomplete Outcome Data	Unclear	There was no description of dropouts or withdrawals.
Selective Reporting	Low	Review authors believe no bias was introduced as all the pre-specified outcomes were adequately reported.
Other sources of bias	High	Three authors were affiliated with CE company and there was no mention of funding, or declaration of conflict of interest. Review authors believe this can introduce bias.
**Overall**	High	

**Table A6:** Risk of bias assessment of Yamano 2013, [13].

Items	Judgement	Specifics
Sequence Generation	Unclear	Quote: “randomly assigned”. Comment: Method of sequence generation was not specified.
Allocation Concealment	Unclear	There was no description of allocation concealment.
Blinding	High	Quote: “protein content, caloric content and color similar to CE” Comment: Even placebo was reported to be prepared in a way to have proteins, calories, and color similar to CEC, placebo can still have different taste and smell compared to CE. Review authors believe this can introduce bias.
Incomplete Outcome Data	Unclear	There was no description of dropouts or withdrawals.
Selective Reporting	Low	Review authors believe no bias was introduced as all the pre-specified outcomes were adequately reported.
Other sources of bias	Unclear	The trial clearly mentioned that it was funded by CE company. In addition, it was clearly mentioned that two of the authors were affiliated with CE company where they were involved with study design and conduct, data analysis, and data interpretation. Review author believes this can introduce bias.
**Overall**	High	

**Table A7:** Risk of bias assessment of Yamano 2015, [5].

Items	Judgement	Specifics
Sequence Generation	Unclear	Quote: “randomly allocated” Comment: Method of sequence generation was not specified.
Allocation Concealment	Unclear	There was no description of allocation concealment.
Blinding	Unclear	Quote: “double-blind”, “blind was successful” Comment: Patients-blinding was checked by test. However, there was no description on the blinding of personnel and outcome assessors.
Incomplete Outcome Data	Low	Quote: “4/50 dropouts where 3 were in placebo arm and 1 in CE arm”. Comment: Review authors believe no bias was introduced.
Selective Reporting	Low	Review authors believe no bias was introduced as all the pre-specified outcomes were adequately reported.
Other sources of bias	Low	The study clearly mentioned it was funded by CE company. However, authors declared no conflict of interest where the funding sponsors had no role in study design, or any part of study design and conduct, or data analysis, or preparation and publication of the study. Review authors believe no risk was introduced.
**Overall**	Unclear	

## Appendix B

**Table B1:** Assessment of quality of trials using Jadad scale.

Items	Nagai 1996, [4]	Azhar 2003	Azhar 2008	Azhar 2013	Konagai 2013, [3]	Yamano 2013	Young 2015
Was the study described as randomized?	0	1	1	1	1	1	1
Was the method used to generate the sequence of randomization described and appropriate?	0	0	0	0	0	0	0
Was the study described as double blind?	0	1	1	1	1	0	1
Was the method of double blinding described and appropriate?	0	0	0	0	0	0	0
Was there a description of withdrawals and dropouts?	1	0	0	1	0	0	1
Deduct 1 point if the method used to generate sequence of randomization was described and it was inappropriate.	0	0	0	0	0	0	0
Deduct 1 point if the study was described as double blind but the method of blinding was inappropriate.	0	−1	−1	−1	−1	0	0
**Total Jadad scores**	**1**	**1**	**1**	**2**	**1**	**1**	**3**
